# Supplementary material for: Botulinum neurotoxin accurately separates tonic vs. phasic transmission and reveals heterosynaptic plasticity rules in Drosophila
Source: eLife. 2022 Aug 22;11:e77924. doi: 10.7554/eLife.77924 (PMC9439677; doi:10.7554/eLife.77924)
Supplement: Supplementary file 1. [file elife-77924-supp1.docx]

**Supplementary Table 1: Characterization of synaptic transmission, growth, and lethality following expression of BoNT-A, -B, and -E.** The genotype, averaged electrophysiological values (with standard error of the mean noted in parentheses), data samples (n), and statistical significance tests are shown.

| **Label** | **Genotype** | **mEPSP amplitude (mV)** | **EPSP amplitude (mV)** | **QC** | **mEPSP frequency (Hz)** | **Rinput (MΩ)** | **Resting potential (mV)** | **n** | **P Value (significance): mEPSP, EPSP, QC, mEPSP freq.** |
| --- | --- | --- | --- | --- | --- | --- | --- | --- | --- |
| wild type | *w^1118^* | 1.01 (±0.037) | 31.46  (±0.841) | 31.61 (±1.166) | 3.25 (±0.128) | 12.01 (±0.136) | 69.14  (±1.115) | 12 | - |
| OK319>BoNT-A | *w*;*OK319-GAL4*/+;*UAS-BoNT-A*/+ | 1.18 (±0.057) | 31.62 (±1.641) | 27.09 (±1.374) | 2.74 (±0.137) | 12.21 (±0.165) | 68.84  (±1.338) | 8 | 0.26 (ns),  0.9999 (ns),  0.26 (ns),  0.08 (ns) |
| OK319>BoNT-B | *w*;*OK319-GAL4*/*UAS-BoNT-B*;+ | 1.48 (±0.129) | 39.34 (±1.858) | 28.01 (±2.561) | 2.27 (±0.203) | 11.81 (±0.278) | 70.47  (±1.454) | 8 | <0.001 (***),  <0.01 (**),  0.43 (ns),  <0.001 (***) |
| OK319>BoNT-E | *w*;*OK319-GAL4*/+;*UAS-BoNT-E*/+ | 1.06 (±0.069) | 34.28  (±2.554 | 32.94 (±2.671) | 3.35 (±0.177) | 11.98 (±0.143 | 71.03  (±1.306) | 9 | 0.92 (ns),  0.50 (ns),  0.93 (ns),  0.95 (ns) |

| **Label** | **Genotype** | **Is Bouton #/M6** | **Ib Bouton #/M6** | **n** | **P Value (significance): Is Bouton, Ib Bouton** |
| --- | --- | --- | --- | --- | --- |
| wild type | *w^1118^* | 23.53  (±0.72) | 28.93  (±0.86) | 15 | - |
| OK319>BoNT-A | *w*;*OK319-GAL4*/+;*UAS-BoNT-A*/+ | 24.33  (±1.80) | 28.33  (±2.33) | 12 | 0.97 (ns),  0.99 (ns), |
| OK319>BoNT-B | *w*;*OK319-GAL4*/*UAS-BoNT-B*;+ | 18.17  (±2.66) | 20.58  (±1.25) | 12 | 0.05 (ns),  <0.01 (**) |
| OK319>BoNT-E | *w*;*OK319-GAL4*/+;*UAS-BoNT-E*/+ | 22.666  (±1.95) | 27.58  (±2.11) | 12 | 0.96 (ns),  0.97 (ns) |

| **Label** | **Transgene insertion** | **Lethality**  **crossed to c155** | **Lethality**  **crossed to OK6** | **Lethality**  **crossed to OK319** | **Lethality**  **crossed to G14** | **Lethality**  **crossed to BG57** |
| --- | --- | --- | --- | --- | --- | --- |
| UAS-BoNT-A | III | Viable | Viable | Viable | Pupal lethal | Viable |
| UAS-BoNT-B | II | Embryonic lethal | Embryonic lethal | Lethal at late 3^rd^ instar | Pupal lethal | Viable |
| UAS-BoNT-C | III | Embryonic lethal | Embryonic lethal | Pupal lethal | Pupal lethal | Sub viable |
| UAS-BoNT-E | III | Embryonic lethal | Viable | Viable | Viable | Viable |
