# Supplementary material for: Botulinum neurotoxin accurately separates tonic vs. phasic transmission and reveals heterosynaptic plasticity rules in Drosophila
Source: eLife. 2022 Aug 22;11:e77924. doi: 10.7554/eLife.77924 (PMC9439677; doi:10.7554/eLife.77924)
Supplement: Supplementary file 2. [file elife-77924-supp2.docx]

**Supplementary Table 2: Absolute values for normalized data and additional statistical details.** The figure and panel, genotype, and conditions are noted. Average values (with standard error of the mean noted in parentheses), data samples (n), and statistical significance tests are shown for all data.

| **Figure** | **Label** | **Genotype** | **mEPSP amplitude (mV)** | **EPSP amplitude (mV)** | **QC** | **mEPSP frequency (Hz)** | **Rinput (MΩ)** | **Resting potential (mV)** | **n** | **P Value (significance): mEPSP, EPSP, QC, mEPSP freq.** |
| --- | --- | --- | --- | --- | --- | --- | --- | --- | --- | --- |
| 1A,B | OK6>ChR2 | *w;OK6-GAL4*/*UAS-ChR2^T159C^*;+ | 0.98 (±0.04) | 27.72 (±0.82) | 28.76 (±1.73) | 3.28 (±0.057) | 12.11 (±0.24) | -67.97 (±1.20) | 10 | - |
| 1A,B | Ib>ChR2 | *w*;*UAS-ChR2^T159C^*/+;*dHb9-GAL4*/+ | 1.05 (±0.06) | 11.53  (±0.78) | 11.31 (±1.10) | 3.23 (±0.14) | 11.71 (±0.270) | -68.36 (±0.82) | 8 | <0.0001 (****), 0.611 (ns),  <0.0001 (****), 0.956 (ns) |
| 1A,B | Is>ChR2 | *w*;*UAS-ChR2^T159C^*/+;*R27E09-GAL4*/+ | 1.04 (±0.06) | 16.52 (±0.97) | 16.36 (±1.34) | 3.14 (±0.14) | 12.22 (±0.16) | -68.56 (±1.10) | 9 | <0.0001 (****), 0.722 (ns),  <0.0001 (****), 0.709 (ns) |
| 1C,D | wild type | *w^1118^* | 1.05 (±0.06) | 35.70 (±1.75) | 34.99 (±3.10) | 3.21 (±0.12) | 12.23 (±0.24) | -69.84 (±1.49) | 8 | - |
| 1C,D | Is>TNT | *w*;*UAS-TNT*/+;*R27E09-GAL4*/+ | 0.935 (±0.05) | 8.407 (±0.62) | 8.976 (±0.40) | 3.010  (±0.13) | 12.144  (±0.34) | -70.515  (±1.13) | 9 | <0.0001 (****), 0.258 (ns),  <0.0001 (****), 0.488 (ns) |
| 1C,D | Ib>TNT | *w*;*UAS-TNT*/+;*dHb9-GAL4*/+ | 0.94  (±0.06) | 24.37  (±1.16) | 26.18  (±1.04) | 3.02  (±0.18) | 12.19  (±0.18) | -67.26  (±0.95) | 8 | <0.0001 (****), 0.301 (ns),  <0.01 (**), 0.568 (ns) |
| 1E,F | wild type | *w^1118^* | 1.00 (±0.06) | 34.36  (±1.57) | 34.78 (±2.45) | 3.28 (±0.07) | 11.92 (±0.17) | -67.32 (±1.66) | 8 | - |
| 1E,F | Is>rpr.hid | *UAS-rpr.hid/w*;+;*R27E09-GAL4*/+ | 0.76 (±0.01) | 9.16 (±0.69) | 11.99 (±0.86) | 1.59 (±0.19) | 11.93 (±0.18) | -71.93 (±1.53) | 9 | <0.0001 (****), 0.646 (ns),  <0.0001 (****), <0.0001 (****) |
| 1E,F | Ib>rpr.hid | *UAS-rpr.hid/w*;+;*dHb9-GAL4*/+ | 0.17 (±0.03) | 0.85 (±0.18) | 2.52 (±0.57) | 0.31 (±0.08) | 12.01 (±0.18) | -70.63 (±0.47) | 10 | <0.0001 (****), 0.607 (ns),  <0.0001 (****), <0.0001 (****) |
| 2D,E | wild type | *w^1118^* | 1.04 (±0.08) | 31.78  (±1.14) | 32.46 (±3.38) | 3.267 (±0.11) | 11.72 (±0.13) | -67.16 (±1.95) | 8 | - |
| 2D,E | OK319>TNT | *w*;*OK319-GAL4*/*UAS-TNT*;+ | 0.94 (±0.04) | 2.25 (±0.43) | 33.97 (±1.93) | 3.13 (±0.11) | 12.17 (±0.16) | -69.91 (±1.28) | 8 | 0.875 (ns), <0.0001 (****),  <0.0001 (****), 0.928 (ns) |
| 2D,E | OK319>BoNT-C | *w*;*OK319-GAL4*/+;*UAS-BoNT-C*/+ | 0.06 (±0.02) | 0.50 (±0.14) | 2.00 (±1.09) | 0.02 (±0.01) | 12.14 (±0.17) | -68.06 (±0.73) | 10 | <0.0001 (****), <0.0001 (****),  <0.0001 (****), <0.0001 (****) |
| 7A-E | wild type  (seed) | *w^1118^* | 0.99 (±0.05) | 32.6 (±2.19) | 34.19 (±3.74) | 3.26 (±0.15) | 12.20 (±0.18) | -70.00 (±1.06) | 10 | - |
| 7A-E | Is>BoNT-C  (seed) | *w*;+;*R27E09-GAL4*/*UAS-BoNT-C* | 0.77 (±0.02) | 9.50  (±0.30) | 12.51 (±0.53) | 2.12 (±0.05) | 12.11 (±0.16) | -70.53 (±0.61) | 17 | - |
| 7A-E | Ib>BoNT-C  (seed) | *w*;+;*dHb9-GAL4*/*UAS-BoNT-C* | 1.34 (±0.04) | 23.65 (±0.48) | 17.92 (±0.62) | 1.51 (±0.05) | 12.05 (±0.14) | -68.60 (±0.65) | 17 | - |
| 7A-E | wild type  (reconstituted) | *w^1118^* | 1.00 (±0.001) | 33.10 (±0.026) | 32.70 (±0.04) | 3.50 (±0.003) | - | - | 10^3^ | - |
| 7A-E | Is>BoNT-C  (reconstituted) | *w*;+;*R27E09-GAL4*/*UAS-BoNT-C* | 0.80 (±0.001) | 9.50  (±0.01) | 12.40 (±0.02) | 2.10 (±0.001) | - | - | 10^3^ | - |
| 7A-E | Ib>BoNT-C  (reconstituted) | *w*;+;*dHb9-GAL4*/*UAS-BoNT-C* | 1.30 (±0.001) | 23.60  (±0.02) | 17.70 (±0.02) | 1.50 (±0.002) | - | - | 10^3^ | - |
| 7A-E | Ib+Is  (reconstituted) | *-* | 1.00  (±0.001) | 33.10 (±0.02) | 30.10 (±0.02) | 3.50 (±0.002) | - | - | 10^3^ | 0.123 (ns), 0.744 (ns),  <0.0001 (****), 0.529 (ns) |
| 8A,B | Is>BoNT-C | *w*;+;*R27E09-GAL4*/*UAS-BoNT-C* | 0.78 (±0.02) | 9.13 (±0.78) | 11.59 (±0.67) | 2.12 (±0.12) | 12.02 (±0.18) | -67.54 (±1.59) | 10 | - |
| 8A,B | Is>TNT | *w*;*UAS-TNT*/+;*R27E09-GAL4*/+ | 0.94 (±0.034) | 8.07 (±0.44) | 8.58 (±0.40) | 2.94 (±0.14) | 12.18 (±0.28) | -69.37 (±1.33) | 9 | <0.05 (*), 0.487 (ns),  <0.05 (*), <0.0001 (****) |
| 8A,B | Is>rpr.hid | *UAS-rpr.hid/w*;+;*R27E09-GAL4*/+ | 0.77  (±0.05) | 14.23 (±0.73) | 18.92 (±0.83) | 2.2  (±0.06) | 11.93  (±0.16) | -68.44 (±1.12) | 12 | 0.988 (ns), <0.0001 (****),  <0.0001 (****), 0.782 (ns) |
| 8C,D | Ib>BoNT-C | *w*;+;*dHb9-GAL4*/*UAS-BoNT-C* | 1.28 (±0.08) | 23.53 (±1.43) | 18.47 (±0.64) | 1.42 (±0.16) | 11.84 (±0.16) | -66.42 (±1.06) | 9 | - |
| 8C,D | Ib>TNT | *w*;*UAS-TNT*/+;*dHb9-GAL4*/+ | 0.94  (±0.06) | 24.37 (±1.16) | 26.18 (±1.04) | 3.02 (±0.18) | 11.69 (±0.26) | -67.58 (±1.71) | 8 | <0.01 (**), 0.801 (ns),  <0.0001 (****), <0.0001 (****) |
| 8C,D | Ib>rpr.hid | *UAS-rpr.hid/w*;+;*dHb9-GAL4*/+ | 0.19  (±0.05) | 0.93 (±0.17) | 2.16 (±0.66) | 0.26 (±0.06) | 11.98 (±0.14) | -68.50 (±1.03) | 10 | <0.0001 (****), <0.0001 (****),  <0.0001 (****), <0.0001 (****) |

| **Figure** | **Label** | **Genotype** | **Muscle** | **mEPSP amplitude (mV)** | **EPSP amplitude (mV)** | **QC** | **mEPSP frequency (Hz)** | **Rinput (MΩ)** | **Resing potential (mV)** | **n** | **P Value (significance): mEPSP, EPSP, QC, mEPSP freq.** |
| --- | --- | --- | --- | --- | --- | --- | --- | --- | --- | --- | --- |
| 8 (extended) | wild type | *w^1118^* | 6 | 1.00  (±0.05) | 32.60 (±2.19) | 34.19 (±3.74) | 3.26  (±0.15) | 12.48 (±0.22) | -68.80 (±1.78) | 10 | - |
| 8 (extended) | Is>BoNT-C | *w*;+;*R27E09-GAL4*/*UAS-BoNT-C* | 6 | 0.78 (±0.02) | 9.13 (±0.78) | 11.59 (±0.67) | 2.12  (±0.12) | 11.61 (±0.14) | -70.62 (±0.65) | 10 | 0.261 (ns), 0.999 (ns),  0.256 (ns), 0.077 (ns) |
| 8 (extended) | Is>TNT | *w*;*UAS-TNT*/+;*R27E09-GAL4*/+ | 6 | 0.94 (±0.03) | 8.07  (±0.44) | 8.59  (±0.40) | 2.94  (±0.14) | 11.95  (±0.15) | -69.43 (±1.27) | 9 | <0.001 (***), <0.01 (**),  0.434 (ns), <0.001 (***) |
| 8 (extended) | Is>rpr.hid | *UAS-rpr.hid/w*;+;*R27E09-GAL4*/+ | 6 | 0.77  (±0.05) | 14.23 (±0.73) | 18.92 (±0.83) | 2.2  (±0.055) | 11.86  (±0.11) | -68.85 (±0.88) | 12 | 0.919 (ns), 0.499 (ns),  0.928 (ns), 0.949 (ns) |
| 8 (extended) | Ib>BoNT-C | *w*;+;*exex-GAL4*/*UAS-BoNT-C* | 6 | 1.28  (±0.08) | 23.53 (±1.43) | 18.47 (±0.64) | 1.42  (±0.16) | 11.77 (±0.17) | -65.65 (±1.35) | 9 | 0.2613 (ns), 0.999 (ns),  0.256 (ns), 0.076 (ns) |
| 8 (extended) | Ib>TNT | *w*;*UAS-TNT*/+;*exex-GAL4*/+ | 6 | 0.94 (±0.06) | 24.37 (±1.16) | 26.18 (±1.04) | 3.02  (±0.18) | 12.10 (±0.19) | -68.40 (±1.31) | 8 | <0.001 (***), <0.01 (**),  0.434 (ns), <0.001 (***) |
| 8 (extended) | Ib>rpr.hid | *UAS-rpr.hid/w*;+;*exex-GAL4*/+ | 6 | 0.19 (±0.05) | 0.93 (±0.17) | 2.16  (±0.66) | 0.26  (±0.06) | 11.81 (±0.16) | -69.71 (±1.05) | 10 | 0.919 (ns), 0.499 (ns),  0.928 (ns), 0.949 (ns) |
| 8 (extended) | wild type | *w^1118^* | 12 | 0.98 (±0.06) | 30.92  (±1.41) | 31.86 (±0.82) | 3.16  (±0.19) | 12.02  (±0.27) | -69.72 (±0.78) | 12 | - |
| 8 (extended) | Is>BoNT-C | *w*;+;*R27E09-GAL4*/*UAS-BoNT-C* | 12 | 0.78 (±0.04) | 9.82 (±0.50) | 12.73 (±0.34) | 2.14 (±0.21) | 12.07 (±0.15) | -70.01 (±0.79) | 8 | <0.05 (*),  <0.0001 (****),  <0.0001 (****),  <0.001 (***) |
| 8 (extended) | Is>TNT | *w*;*UAS-TNT*/+;*R27E09-GAL4*/+ | 12 | 0.95 (±0.06) | 10.50  (±0.76) | 11.13 (±0.39) | 2.76  (±0.14) | 12.3 (±0.244) | -69.02 (±1.21) | 8 | 0.957 (ns),  <0.0001 (****),  <0.0001 (****),  0.377 (ns) |
| 8 (extended) | Is>rpr.hid | *UAS-rpr.hid/w*;+;*R27E09-GAL4*/+ | 12 | 0.76 (±0.03) | 12.66  (±0.62) | 16.72 (±0.52) | 2.08  (±0.12) | 12.17 (±0.15) | -69.29 (±1.45) | 9 | <0.05 (*),  <0.0001 (****),  <0.0001 (****),  <0.001 (***) |
| 8 (extended) | wild type | *w^1118^* | 4 | 1.01 (±0.05) | 27.69  (±1.35) | 28.25 (±2.08) | 3.13 (±0.09) | 12.05  (±0.11) | -69.48 (±0.94) | 11 | - |
| 8 (extended) | Is>BoNT-C | *w*;+;*R27E09-GAL4*/*UAS-BoNT-C* | 4 | 0.75 (±0.03) | 13.25 (±0.66) | 17.95 (±1.11) | 2.19 (±0.08) | 12.32 (±0.21) | -70.02 (±0.96) | 12 | <0.001 (***),  <0.0001 (****),  <0.0001 (****),  <0.0001 (****) |
| 8 (extended) | Is>TNT | *w*;*UAS-TNT*/+;*R27E09-GAL4*/+ | 4 | 1.04 (±0.05) | 13.55 (±0.64) | 13.41 (±1.09) | 2.84 (±0.15) | 11.91 (±0.18) | -67.15 (±0.79) | 12 | 0.949 (ns),  <0.0001 (****),  <0.0001 (****),  0.2210 (ns) |
| 8 (extended) | Is>rpr.hid | *UAS-rpr.hid/w*;+;*R27E09-GAL4*/+ | 4 | 0.79 (±0.03) | 16.24  (±0.99) | 20.89 (±1.28) | 2.07 (±0.08) | 11.68 (±0.19) | -69.42 (±0.83) | 12 | <0.01 (**),  <0.0001 (****),  <0.01 (**),  <0.0001 (****) |

| **Figure** | **Label** | **Genotypes** | **NMJ** | **Bouton #/M6** | **n** | **BRP puncta #/M6** | **BRP puncta intensity/M6 (%WT)** | **n** | **P Value (significance): Bouton#, BRP#, BRP intensity** |
| --- | --- | --- | --- | --- | --- | --- | --- | --- | --- |
| 3A,B | wild type | *w^1118^* | Ib | 28.50 (±1.21) | 16 | 217.33 (±9.61) | 100  (±4.93) | 12 | - |
| 3A,B | OK319>TNT | *w*;*OK319-GAL4*/*UAS-TNT*;+ | Ib | 23.09 (±0.77) | 11 | 192.58 (±4.56) | 98.13  (±4.41) | 12 | <0.05 (*),  <0.05 (*),  0.933 (ns) |
| 3A,B | OK319>BoNT-C | *w*;*OK319-GAL4*/+;*UAS-BoNT-C*/+ | Ib | 27.92 (±2.14) | 12 | 210.58 (±5.71) | 97.79  (±3.45) | 12 | 0.944 (ns),  0.719 (ns),  0.908 (ns) |
| 3A,B | wild type | *w^1118^* | Is | 23.19 (±0.56) | 16 | 93.58 (±3.70) | 100  (±3.93) | 12 | - |
| 3A,B | OK319>TNT | *w*;*OK319-GAL4*/*UAS-TNT*;+ | Is | 28.55 (±0.95) | 11 | 111.17 (±6.24) | 102.97 (±4.75) | 12 | <0.05 (*),  <0.05 (*),  0.810 (ns) |
| 3A,B | OK319>BoNT-C | *w*;*OK319-GAL4*/+;*UAS-BoNT-C*/+ | Is | 23.58 (±2.23) | 12 | 99.50 (±3.86) | 93.06  (±2.63) | 12 | 0.968 (ns),  0.586 (ns),  0.352 (ns) |

| **Figure** | **Label** | **Genotypes** | **T-bar length (nm)** | **Active zone length (nm)** | **Vesicle density (#/µm^2^)** | **n** | **P Value (significance): T-bar length, AZ length, Vesicle density** |
| --- | --- | --- | --- | --- | --- | --- | --- |
| 3C,D | wild type | *w^1118^* | 122.29 (±2.85) | 584.78 (±14.48) | 171.32  (±5.69) | 18 | - |
| 3C,D | OK319>TNT | *w*;*OK319-GAL4*/*UAS-TNT*;+ | 122.76 (±3.61) | 522.93 (±14.94) | 183.51  (±4.48) | 20 | <0.05 (*),  <0.05 (*),  0.195 (ns) |
| 3C,D | OK319>BoNT-C | *w*;*OK319-GAL4*/+;*UAS-BoNT-C*/+ | 123.07 (±3.64) | 555.47 (±17.40) | 168.51  (±5.76) | 19 | 0.944 (ns),  0.719 (ns),  0.904 (ns) |

| **Figure** | **Label** | **Genotypes** | **NMJ** | **GluRIIA puncta intensity**  **(% wild type)** | **GluRIIB puncta intensity**  **(% wild type)** | **GluRIID puncta intensity**  **(% wild type)** | **n** | **P Value (significance): GluRIIA, GluRIIB, GluRIID** |
| --- | --- | --- | --- | --- | --- | --- | --- | --- |
| 4B | wild type | *w^1118^* | Ib | 100  (±3.70) | 100  (±4.09) | 100  (±2.56) | 16 | - |
| 4B | wild type | *w^1118^* | Is | 67.28  (±2.78) | 121.53  (±7.87) | 94.26  (±3.84) | 15 | <0.0001 (****),  <0.05 (*),  0.218 (ns) |
| 4E | OK319>BoNT-C | *w*;*OK319-GAL4*/+;*UAS-BoNT-C*/+ | Ib | 100  (±3.12) | 100  (±2.60) | 100  (±4.38) | 14 | - |
| 4E | OK319>BoNT-C | *w*;*OK319-GAL4*/+;*UAS-BoNT-C*/+ | Is | 68.25  (±3.88) | 83.73  (±2.18) | 93.89  (±4.31) | 14 | <0.0001 (****),  <0.01 (**),  0.329 (ns) |

| **Figure** | **Label** | **Genotypes** | **NMJ** | **Transmission** | **Quantal size (ΔF/F)** | **n** | **P Value (significance):** |
| --- | --- | --- | --- | --- | --- | --- | --- |
| 5B | Is>BoNT-C | *w*;*MHC>GCaMP8f*/+;*R27E09-GAL4*/*UAS-BoNT-C* | Ib | spontaneous | 0.029  (0.002) | 5 | - |
| 5B | Is>BoNT-C | *w*;*MHC>GCaMP8f*/+;*R27E09-GAL4*/*UAS-BoNT-C* | Is | spontaneous | 0.004  (0.001) | 5 | <0.0001 (****) |
| 5C | Is>BoNT-C | *w*;*MHC>GCaMP8f*/+;*R27E09-GAL4*/*UAS-BoNT-C* | Ib | evoked | 0.326  (±0.018) | 5 | - |
| 5C | Is>BoNT-C | *w*;*MHC>GCaMP8f*/+;*R27E09-GAL4*/*UAS-BoNT-C* | Is | evoked | 0.013  (±0.001) | 5 | <0.0001 (****) |
| 5E | Ib>BoNT-C | *w*;*MHC>GCaMP8f*/+;*dHb9-GAL4*/*UAS-BoNT-C* | Ib | spontaneous | 0.005  (±0.001) | 5 | - |
| 5E | Ib>BoNT-C | *w*; *MHC>GCaMP8f*/+;*dHb9-GAL4*/*UAS-BoNT-C* | Is | spontaneous | 0.046  (±0.004) | 5 | <0.0001 (****) |
| 5F | Ib>BoNT-C | *w*;*MHC>GCaMP8f*/+;*dHb9-GAL4*/*UAS-BoNT-C* | Ib | evoked | 0.013  (±0.001) | 5 | - |
| 5F | Ib>BoNT-C | *w*; *MHC>GCaMP8f*/+;*dHb9-GAL4*/*UAS-BoNT-C* | Is | evoked | 0.475  (±0.026) | 5 | <0.0001 (****) |
| S2D | wild type (L1) | *w*;*MHC>GCaMP8f*/+;+ | Ib | spontaneous | 0.013  (±0.001) | 5 | - |
| S2D | OK319>BoNT-C (L1) | *w*;*MHC>GCaMP8f*/*OK319-GAL4*;+/*UAS-BoNT-C* | Ib | spontaneous | 0.001  (±0.000) | 5 | <0.0001 (****) |
| S2D | wild type (L1) | *w*;*MHC>GCaMP8f*/+;+ | Is | spontaneous | 0.018  (±0.001) | 5 | - |
| S2D | OK319>BoNT-C (L1) | *w*;*MHC>GCaMP8f*/*OK319-GAL4*;+/*UAS-BoNT-C* | Is | spontaneous | 0.002  (±0.000) | 5 | <0.0001 (****) |
| S2G | Is>BoNT-C (L1) | *w*;*MHC>GCaMP8f*/+;*R27E09-GAL4*/*UAS-BoNT-C* | Ib | spontaneous | 0.030  (±0.001) | 5 | - |
| S2G | Ib>BoNT-C (L1) | *w*;*MHC>GCaMP8f*/+;*dHb9-GAL4*/*UAS-BoNT-C* | Ib | spontaneous | 0.004  (±0.000) | 5 | <0.0001 (****) |
| S2G | Is>BoNT-C (L1) | *w*;*MHC>GCaMP8f*/+;*R27E09-GAL4*/*UAS-BoNT-C* | Is | spontaneous | 0.004  (±0.001) | 5 | - |
| S2G | Ib>BoNT-C (L1) | *w*;*MHC>GCaMP8f*/+;*dHb9-GAL4*/*UAS-BoNT-C* | Is | spontaneous | 0.043  (±0.002) | 5 | <0.0001 (****) |

| **Figure** | **Label** | **Genotype** | **Muscle** | **Is Bouton #/NMJ** | **Ib Bouton #/NMJ** | **n** | **P Value (significance): Is Bouton, Ib Bouton** |
| --- | --- | --- | --- | --- | --- | --- | --- |
| 6A-D | wild type | *w^1118^* | 6 | 23.53  (±0.72) | 28.33  (±0.86) | 15 | - |
| 6A-D | Is>BoNT-C | *w*;+;*R27E09-GAL4*/*UAS-BoNT-C* | 6 | 24.33  (±1.80) | 25.56  (±0.79) | 16 | 0.993 (ns),  0.999 (ns), |
| 6A-D | Is>TNT | *w*;*UAS-TNT*/+;*R27E09-GAL4*/+ | 6 | 27.83  (±1.65) | 27.67  (±1.52) | 12 | <0.05 (*),  0.811 (ns) |
| 6A-D | Is>rpr.hid | *UAS-rpr.hid/w*;+;*R27E09-GAL4*/+ | 6 | 31.73  (±0.83) | 31.73  (±0.83) | 11 | <0.0001 (****),  <0.001 (***) |
| 6F-I | wild type | *w^1118^* | 6 | 23.23  (±0.48) | 27.15  (±0.68) | 13 | - |
| 6F-I | Ib>BoNT-C | *w*;+;*dHb9-GAL4*/*UAS-BoNT-C* | 6 | 23.69  (±0.71) | 25.63  (±0.65) | 16 | 0.999 (ns),  0.999 (ns), |
| 6F-I | Ib>TNT | *w*;*UAS-TNT*/+;*dHb9-GAL4*/+ | 6 | 22.09  (±1.56) | 21.08  (±0.81) | 11 | 0.982 (ns),  <0.01 (**) |
| 6F-I | Ib>rpr.hid | *UAS-rpr.hid/w*;+;*dHb9-GAL4*/+ | 6 | 0.28  (±0.11) | 5.44  (±0.85) | 25 | <0.0001 (****),  <0.0001 (****) |

| **Figure** | **Label** | **Genotype** | **Muscle** | **Is Bouton #/NMJ** | **Significant Change (%WT)** | **Ib Bouton #/NMJ** | **Significant Change (%WT)** | **n** | **P Value (significance): Is Bouton, Ib Bouton** |
| --- | --- | --- | --- | --- | --- | --- | --- | --- | --- |
| 6  (extended) | wild type | *w^1118^* | 6 | 23.53  (±0.72) | - | 28.33  (±0.86) | - | 15 | - |
| 6  (extended) | Is>BoNT-C | *w*;+;*R27E09-GAL4*/*UAS-BoNT-C* | 6 | 24.33  (±1.80) | - | 25.56  (±0.79) | - | 16 | 0.993 (ns),  0.999 (ns), |
| 6  (extended) | Is>TNT | *w*;*UAS-TNT*/+;*R27E09-GAL4*/+ | 6 | 27.83  (±1.65) | ↑24 | 27.67  (±1.52) | - | 12 | <0.05 (*),  0.811 (ns) |
| 6  (extended) | Is>rpr.hid | *UAS-rpr.hid/w*;+;*R27E09-GAL4*/+ | 6 | 31.73  (±0.83) | ↓100 | 31.73  (±0.83) | ↑18 | 11 | <0.0001 (****),  <0.001 (***) |
| 6  (extended) | Ib>BoNT-C | *w*;+;*exex-GAL4*/*UAS-BoNT-C* | 6 | 23.69  (±0.71) | - | 25.63  (±0.65) | - | 16 | 0.999 (ns),  0.999 (ns), |
| 6  (extended) | Ib>TNT | *w*;*UAS-TNT*/+;*exex-GAL4*/+ | 6 | 22.09  (±1.56) | - | 21.08  (±0.81) | ↓22 | 11 | 0.982 (ns),  <0.01 (**) |
| 6  (extended) | Ib>rpr.hid | *UAS-rpr.hid/w*;+;*exex-GAL4*/+ | 6 | 0.28  (±0.11) | ↓99 | 5.44  (±0.85) | ↓94 | 25 | <0.0001 (****),  <0.0001 (****) |
| 6  (extended) | wild type | *w^1118^* | 7 | 12.63  (±1.29) | - | 18.81  (±1.41) | - | 15 | - |
| 6  (extended) | Is>BoNT-C | *w*;+;*R27E09-GAL4*/*UAS-BoNT-C* | 7 | 12.13  (±0.53) | - | 19.88  (±0.58) | - | 16 | 0.879 (ns),  0.6678 (ns), |
| 6  (extended) | Is>TNT | *w*;*UAS-TNT*/+;*R27E09-GAL4*/+ | 7 | 18.92  (±1.51) | ↑49 | 18.58  (±2.10) | - | 12 | <0.01 (**),  0.993 (ns) |
| 6  (extended) | Is>rpr.hid | *UAS-rpr.hid/w*;+;*R27E09-GAL4*/+ | 7 | 0  (±0) | ↓100 | 22.64  (±1.73) | ↑20 | 11 | <0.0001 (****),  0.991 (ns) |
| 6  (extended) | Ib>BoNT-C | *w*;+;*exex-GAL4*/*UAS-BoNT-C* | 7 | 12.13  (±0.41) | - | 19.38  (±0.78) | - | 16 | 0.879 (ns),  0.889 (ns), |
| 6  (extended) | Ib>TNT | *w*;*UAS-TNT*/+;*exex-GAL4*/+ | 7 | 13.73  (±1.51) | - | 12.82  (±1.23) | ↓30 | 11 | 0.814 (ns),  <0.05 (*) |
| 6  (extended) | Ib>rpr.hid | *UAS-rpr.hid/w*;+;*exex-GAL4*/+ | 7 | 0  (±0) | ↓99 | 0.44  (±0.18) | ↓99 | 25 | <0.0001 (****),  <0.0001 (****) |
| 6  (extended) | wild type | *w^1118^* | 12 | 23.75  (±0.993) | - | 18.08  (±0.87) | - | 12 | - |
| 6  (extended) | Is>BoNT-C | *w*;+;*R27E09-GAL4*/*UAS-BoNT-C* | 12 | 24.64  (±2.76) | - | 17.82  (±2.03) | - | 11 | 0.993 (ns),  0.999 (ns), |
| 6  (extended) | Is>TNT | *w*;*UAS-TNT*/+;*R27E09-GAL4*/+ | 12 | 18.92  (±1.51) | ? | 18.58  (±2.10) | - | 12 | <0.05 (*),  0.999 (ns) |
| 6  (extended) | Is>rpr.hid | *UAS-rpr.hid/w*;+;*R27E09-GAL4*/+ | 12 | 0  (±0) | ↓100 | 26.82  (±1.48) | ↑48 | 11 | <0.0001 (****),  <0.001 (***) |
| 6  (extended) | wild type | *w^1118^* | 13 | 15.50  (±0.48) | - | 15.33  (±0.41) | - | 12 | - |
| 6  (extended) | Is>BoNT-C | *w*;+;*R27E09-GAL4*/*UAS-BoNT-C* | 13 | 15.55  (±0.62) | - | 15.91  (±0.41) | - | 11 | 0.999 (ns),  0.793 (ns), |
| 6  (extended) | Is>TNT | *w*;*UAS-TNT*/+;*R27E09-GAL4*/+ | 13 | 21.83  (±0.81) | ↑40 | 14.83  (±0.53) | - | 12 | <0.0001 (****),  0.843 (ns) |
| 6  (extended) | Is>rpr.hid | *UAS-rpr.hid/w*;+;*R27E09-GAL4*/+ | 13 | 0  (±0) | ↓100 | 20.73  (±0.74) | ↑35 | 11 | <0.0001 (****),  <0.0001 (****) |
| 6  (extended) | wild type | *w^1118^* | 4 | 22.58  (±0.99) | - | 18.67  (±1.39) | - | 12 | - |
| 6  (extended) | Is>BoNT-C | *w*;+;*R27E09-GAL4*/*UAS-BoNT-C* | 4 | 24.64  (±2.33) | - | 20.91  (±1.46) | - | 11 | 0.872 (ns),  0.837 (ns), |
| 6  (extended) | Is>TNT | *w*;*UAS-TNT*/+;*R27E09-GAL4*/+ | 4 | 28.83  (±1.17) | ↑27 | 19.67  (±1.66) | - | 12 | <0.05 (*),  0.995 (ns) |
| 6  (extended) | Is>rpr.hid | *UAS-rpr.hid/w*;+;*R27E09-GAL4*/+ | 4 | 0  (±0) | ↓100 | 27.27  (±1.24) | ↑45 | 11 | <0.0001 (****),  <0.01 (**) |

| **Figure** | **Label** | **Genotypes** | **Ib vGlut**  **puncta intensity**  **(% wild type)** | **Is vGlut**  **puncta intensity**  **(% wild type)** | **n** | **P Value (significance): Ib vGlut, Is vGlut** |
| --- | --- | --- | --- | --- | --- | --- |
| S3D | wild type | *w^1118^* | 100  (±3.59) | 100  (±3.47) | 10 | - |
| S3D | D42>vGlut^-/-^ | *w;vGlut^SS1^*/*B3RT-vGlut-B3RT*;*UAS-B3*/*D42-GAL4* | 61.49  (±2.33) | 61.49  (±3.60) | 10 | <0.0001 (****),  <0.0001 (****) |
| S3D | Is>vGlut^-/-^ | *w;vGlut^SS1^*/*B3RT-vGlut-B3RT*;*UAS-B3*/*R7E09-GAL4* | 94.24  (±3.36) | 68.46  (±2.71) | 11 | 0.384 (ns),  <0.0001 (****) |
| S3D | Ib>vGlut^-/-^ | *w;vGlut^SS1^*/*B3RT-vGlut-B3RT*;*UAS-B3*/*dHB9-GAL4* | 63.36  (±2.33) | 98.21  (±3.77) | 11 | <0.0001 (****),  0.965 (ns) |

| **Figure** | **Label** | **Genotype** | **mEPSP amplitude (mV)** | **EPSP amplitude (mV)** | **QC** | **mEPSP frequency (Hz)** | **Rinput (MΩ)** | **Resting potential (mV)** | **n** | **P Value (significance): mEPSP, EPSP, QC, mEPSP freq.** |
| --- | --- | --- | --- | --- | --- | --- | --- | --- | --- | --- |
| S3B | wild type | *w^1118^* | 0.96 (±0.04) | 34.12 (±1.70) | 36.77 (±3.16) | 3.09 (±0.15) | 12.33 (±0.17) | -69.77 (±1.04) | 11 | - |
| S3B | Is>vGlut^-/-^ | *w;vGlut^SS1^*/*B3RT-vGlut-B3RT*;*UAS-B3*/*R7E09-GAL4* | 0.93 (±0.03) | 28.36 (±1.23) | 30.40 (±0.73) | 2.66  (±0.09) | 12.28  (±0.24) | 71.65  (±0.75) | 11 | <0.0001 (****), 0.258 (ns),  <0.0001 (****), 0.488 (ns) |
| S3B | Ib>vGlut^-/-^ | *w;vGlut^SS1^*/*B3RT-vGlut-B3RT*;*UAS-B3*/*dHB9-GAL4* | 1.11 (±0.03) | 31.88  (±2.12) | 28.56 (±1.30) | 2.51 (±0.15) | 11.52  (±0.23) | -68.94  (±1.12) | 8 | <0.0001 (****), 0.301 (ns),  <0.01 (**), 0.568 (ns) |
| S4B | wild type  (electrical) | *w^1118^* | 1.092 (±0.09) | 32.29 (±0.69) | 28.28 (±2.31) | 3.09 (±0.15) | 11.72 (±0.21) | -68.55 (±1.45) | 11 | - |
| S4B | OK6>ChR2  (electrical) | *w;OK6-GAL4*/*UAS-ChR2^T159C^*;+ | 0.98 (±0.04) | 27.72 (±0.82) | 28.76 (±1.73) | 2.68 (±0.16) | 12.03  (±0.17) | 69.93  (±1.16) | 10 | <0.0001 (****), 0.258 (ns),  <0.0001 (****), 0.488 (ns) |
| S4B | OK6>ChR2  (optical) | *w;OK6-GAL4*/*UAS-ChR2^T159C^*;+ | 0.98 (±0.04) | 23.63 (±0.77) | 24.41 (±1.23) | 2.68 (±0.16) | 12.03  (±0.17) | 69.93  (±1.16) | 10 | <0.0001 (****), 0.301 (ns),  <0.01 (**), 0.568 (ns) |
| S4D | Is>BoNT-C  (electrical) | *w*;+;*R27E09-GAL4*/*UAS-BoNT-C* | 0.76 (±0.05) | 9.71 (±0.48) | 13.22 (±0.93) | 2.65 (±0.12) | 11.68  (±0.18) | 68.43  (±0.97) | 10 | <0.0001 (****), 0.258 (ns),  <0.0001 (****), 0.488 (ns) |
| S4D | Ib>ChR2  (optical) | *w*;*UAS-ChR2^T159C^*/+;*dHb9-GAL4*/+ | 1.046 (±0.06) | 7.87  (±0.47) | 7.642 (±0.54) | 2.86 (±0.18) | 11.92  (±0.23) | -70.68  (±0.91) | 8 | <0.0001 (****), 0.301 (ns),  <0.01 (**), 0.568 (ns) |
| S4F | Ib>BoNT-C  (electrical) | *w*;+;*dHb9-GAL4*/*UAS-BoNT-C* | 1.242 (±0.11) | 23.23 (±1.67) | 19.99 (±2.43) | 2.06  (±0.09) | 11.74  (±0.22) | 68.38  (±1.75) | 9 | <0.0001 (****), 0.258 (ns),  <0.0001 (****), 0.488 (ns) |
| S4F | Is>ChR2  (optical) | *w*;*UAS-ChR2^T159C^*/+;*R27E09-GAL4*/+ | 1.04 (±0.06) | 16.15  (±0.74) | 16.00 (±1.17) | 2.74 (±0.17) | 12.08  (±0.16) | -68.46  (±1.16) | 9 | <0.0001 (****), 0.301 (ns),  <0.01 (**), 0.568 (ns) |

| **Figure** | **Label** | **Genotype** | **Muscle 6/7 Is Bouton #/NMJ** | **Muscle 6/7 Ib Bouton #/NMJ** | **n** | **P Value (significance): Is Bouton, Ib Bouton** |
| --- | --- | --- | --- | --- | --- | --- |
| S5A,B | wild type | *w^1118^* | 34.94  (±4.07) | 53.88  (±3.19) | 17 | - |
| S5A,B | Is>rpr.hid | *UAS-rpr.hid/w*;+;*R27E09-GAL4*/+ | 0.91  (±0.21) | 61.91  (±2.51) | 11 | <0.0001 (****),  <0.05 (*) |
| S5A,B | Ib>rpr.hid | *UAS-rpr.hid/w*;+;*dHb9-GAL4*/+ | 0.22  (±0.09) | 5.89  (±1.05) | 27 | <0.0001 (****),  <0.0001 (****) |
| S5E,F | Ib>rpr.hid  (total loss) | *UAS-rpr.hid/w*;+;*dHb9-GAL4*/+ | 0  (±0) | 1.10  (±0.28) | 10  (37%) | - |
| S5E,F | Ib>rpr.hid  (partial loss) | *UAS-rpr.hid/w*;+;*dHb9-GAL4*/+ | 0.35  (±0.15) | 8.71  (±1.22) | 17  (63%) | - |

| **Figure** | **Label** | **Genotypes** | **GluRIIA puncta intensity**  **(% wild type)** | **GluRIID puncta intensity**  **(% wild type)** | **GluRIIA puncta size (µm^2^)** | **GluRIID puncta size (µm^2^)** | **Bouton #** | **NMJ area (µm^2^)** | **n** | **P Value (significance): IIA intensity, IID intensity, IIA size, IID size, Bouton #, NMJ area** |
| --- | --- | --- | --- | --- | --- | --- | --- | --- | --- | --- |
| S6B | wild type (L1) | *w^1118^* | 100  (±4.61) | 100  (±5.73) | 0.22  (±0.01) | 0.20  (±0.01) | 20.2  (±1.39) | 49.24  (±3.09) | 5 | - |
| S6B | OK319>BoNT-C (L1) | *w*;*OK319-GAL4*/+;*UAS-BoNT-C*/+ | 98.33  (±9.28) | 89.95  (±2.90) | 0.20  (±0.01) | 0.22  (±0.01) | 19.6  (±1.43) | 53.99  (±0.96) | 5 | 0.190 (ns), 0.123 (ns), 0.172 (ns),  0.194 (ns), 0.771 (ns), 0.179 (ns) |
| S6D | wild type (L2) | *w^1118^* | 100  (±4.58) | 100  (±3.66) | 0.27  (±0.02) | 0.28  (±0.03) | 34.80  (±1.39) | 103.10  (±8.37) | 5 | - |
| S6D | OK319>BoNT-C (L2) | *w*;*OK319-GAL4*/+;*UAS-BoNT-C*/+ | 105.10  (±14.94) | 93.01  (±4.31) | 0.24  (±0.03) | 0.26  (±0.02) | 32.5  (±1.54) | 103.2  (±4.94) | 5 | 0.211 (ns), 0.097 (ns), 0.452 (ns),  0.568 (ns), 0.306 (ns), 0.993 (ns) |

| **Figure** | **Label** | **Genotype** | **mEPSC amplitude (nA)** | **EPSC amplitude (nA)** | **QC** | **mEPSC frequency (Hz)** | **Rinput (MΩ)** | **Resting potential (mV)** | **n** | **P Value (significance): mEPSP, EPSP, QC, mEPSP freq.** |
| --- | --- | --- | --- | --- | --- | --- | --- | --- | --- | --- |
| S7A-E | wild type  (seed) | *w^1118^* | 44.88  (±1.22) | 0.51  (±0.02) | 88.69  (±3.54) | 3.86  (±0.06) | 12.20 (±0.18) | -70.00 (±1.06) | 15 | - |
| S7A-E | Is>BoNT-C  (seed) | *w*;+;*R27E09-GAL4*/*UAS-BoNT-C* | 14.47  (±0.53) | 0.39  (±0.01) | 37.73  (±1.77) | 2.49  (±0.08) | 12.11 (±0.16) | -70.53 (±0.61) | 15 | - |
| S7A-E | Ib>BoNT-C  (seed) | *w*;+;*dHb9-GAL4*/*UAS-BoNT-C* | 28.40  (±0.98) | 0.66  (±0.02) | 43.53  (±2.15) | 1.32  (±0.03) | 12.05 (±0.14) | -68.60 (±0.65) | 15 | - |
| S7A-E | wild type  (reconstituted) | *w^1118^* | 42.99  (±0.04) | 0.48  (±0.00) | 89.42  (±0.12) | 3.81  (±0.01) | - | - | 10^3^ | - |
| S7A-E | Is>BoNT-C  (reconstituted) | *w*;+;*R27E09-GAL4*/*UAS-BoNT-C* | 14.46  (±0.02) | 0.38  (±0.01) | 37.35  (±0.05) | 2.48  (±0.01) | - | - | 10^3^ | - |
| S7A-E | Ib>BoNT-C  (reconstituted) | *w*;+;*dHb9-GAL4*/*UAS-BoNT-C* | 28.44  (±0.03) | 0.66  (±0.01) | 43.06  (±20.06) | 1.31  (±0.03) | - | - | 10^3^ | - |
| S7A-E | Ib+Is  (reconstituted) | *-* | 42.91  (±0.04) | 0.48  (±0.01) | 88.04  (±0.09) | 3.80  (±0.01) | - | - | 10^3^ | 0.212 (ns), 0.086 (ns),  <0.013 (*), 0.120 (ns) |
